# Leveraging pre-trained machine learning models for islet quantification in type 1 diabetes

**DOI:** 10.1016/j.jpi.2024.100406

**Published:** 2024-11-08

**Authors:** Sanghoon Kang, Jesus D. Penaloza Aponte, Omar Elashkar, Juan Francisco Morales, Nicholas Waddington, Damon G. Lamb, Huiwen Ju, Martha Campbell-Thompson, Sarah Kim

**Affiliations:** a Department of Pharmaceutics, Center for Pharmacometrics and Systems Pharmacology, Intelligent Critical Care Center, College of Pharmacy, University of Florida, Orlando, FL, USA; b Department of Pathology, Immunology, and Laboratory Medicine, Diabetes Institute, College of Medicine, University of Florida, Gainesville, FL, USA; c Department of Neuroscience, McKnight Brain Institute, University of Florida, Gainesville, FL, USA; d Departments of Psychiatry, Neuroscience, Biomedical Engineering, McKnight Brain Institute, College of Medicine, University of Florida, Gainesville, FL, USA; e Malcom Randall VAMC, Gainesville, FL, USA; f NVIDIA Corporation, Santa Clara, CA, USA

**Keywords:** Type 1 diabetes, Islet heterogeneity, Machine learning, Digital pathology, Whole slide images

## Abstract

Human islets display a high degree of heterogeneity in terms of size, number, architecture, and endocrine cell-type compositions. An ever-increasing number of immunohistochemistry-stained whole slide images (WSIs) are available through the online pathology database of the Network for Pancreatic Organ donors with Diabetes (nPOD) program at the University of Florida (UF). We aimed to develop an enhanced machine learning-assisted WSI analysis workflow to utilize the nPOD resource for analysis of endocrine cell heterogeneity in the natural history of type 1 diabetes (T1D) in comparison to donors without diabetes. To maximize usability, the user-friendly open-source software QuPath was selected for the main interface. The WSI data were analyzed with two pre-trained machine learning models (i.e., Segment Anything Model (SAM) and QuPath's pixel classifier), using the UF high-performance-computing cluster, HiPerGator. SAM was used to define precise endocrine cell and cell grouping boundaries (with an average quality score of 0.91 per slide), and the artificial neural network-based pixel classifier was applied to segment areas of insulin- or glucagon-stained cytoplasmic regions within each endocrine cell. An additional script was developed to automatically count CD3+ cells inside and within 20 μm of each islet perimeter to quantify the number of islets with inflammation (i.e., CD3+ T-cell infiltration). Proof-of-concept analysis was performed to test the developed workflow in 12 subjects using 24 slides. This open-source machine learning-assisted workflow enables rapid and high throughput determinations of endocrine cells, whether as single cells or within groups, across hundreds of slides. It is expected that the use of this workflow will accelerate our understanding of endocrine cell and islet heterogeneity in the context of T1D endotypes and pathogenesis.

## Introduction

Type 1 diabetes (T1D) is an autoimmune disease characterized by the destruction of insulin-producing pancreatic islet β-cells. Islets of Langerhans are clusters of cells in the pancreas that secrete hormones, including insulin from beta cells and glucagon from alpha cells, to regulate blood sugar levels.^1^ Clinical onset of T1D occurs when there is insufficient functional β-cell mass resulting in hyperglycemia. As T1D develops, loss of β-cells occurs as well as inflammation of the islets (CD3+ T-cell infiltration).^2^ To aid in concordance between studies of T1D histopathology, a consensus definition of CD3+ T-cell infiltration was established in 2013: T1D was defined by the presence of ≥3 islets without β-cells (also called pseudoatrophic islets) and CD3+ T-cell infiltration as the presence of ≥3 islets with ≥15 CD45+ cells within or touching the perimeter of an islet.^3^ Further studies expanded the definition to include islets with ≥6 infiltrating CD3+ cells as insulitic.^2^

Several challenges have limited research on the pathogenesis of T1D, attributed to the following factors: first, a high degree of heterogeneity among pancreas islets (i.e., the number of each endocrine cell type and total endocrine cells within each islet). Second, manual analysis for detecting and quantifying islets is time-consuming given the large number of islets in each whole slide image (WSI).^4^ Third, studying the histopathology of T1D in living subjects is not feasible due to severe complications associated with pancreatic biopsy.^5^

Computational pathology is a growing field in clinical and research pathology that is expected to hasten understanding of disease pathogenesis using microscopic tissue sections.^6^^,^^7^ Manual histopathological analysis can be time consuming and subject to individual bias leading to limited reproducibility and consistency between studies due to the heterogeneity of human pancreas.^8^, ^9^, ^10^ Additionally, it is challenging to find qualified pathologists to perform manual histopathological analysis. In contrast, WSI analysis offers numerous advantages, particularly the ability to assess changes across a very large number of subjects and tissue sections. Recently, WSI analysis using ImageJ or QuPath software has been applied in several studies of endocrine cell heterogeneity in the context of T1D pathogenesis.^11^, ^12^, ^13^, ^14^ The Network for Pancreatic Organ Donors with Diabetes (nPOD) program^15^ provides online access to numerous human pancreatic WSI of various T1D risk-levels, ranging from donors with no diabetes (ND), ND but islet autoantibody-positive (AAb+) individuals, and those with T1D ranging in durations from onset to ≥50 years. Additionally, multiple WSI are often available from three regions of the pancreas: head, body, and tail. The serial sections are stained by standard hematoxylin and eosin (H&E) and also multiplex immunohistochemistry (IHC) to differentially label islet endocrine cells or CD3+ lymphocytes.^16^ Corresponding donor demographics, clinical history, and laboratory assessments are also collected. We aimed to develop an enhanced machine learning-assisted WSI analysis workflow to utilize the online pathology nPOD resource for the analysis of endocrine cell heterogeneity in the natural history of T1D with open-source software.

## Technical background and objectives

Deep learning-based segmentation and object detection models have substantially improved WSI analysis more accurate, rapid, and reproducible. Machine learning methods such as extreme gradient boosting (XGBoost), convolutional neural networks (CNNs), and transfer learning have been utilized for analyzing bioimages for detection, segmentation, and classification. The transfer learning was utilized to improve the classification performance for WSI patches,^17^ and it also enhanced the performance of CNN networks in the detection and classification of breast cancer.^18^ Additionally, novel data augmentation techniques were suggested to further improve the CNN-based model for breast cancer detection and classification.^19^ A CNN was also used to classify kidney cancer subtype^20^ and to detect and diagnose prostate cancer.^21^ Alternatively, a random forest was harnessed to diagnose breast cancer.^22^

Few studies have focused on analyzing the pathology of T1D: large-scale image analysis of endocrine cells and immune cells through semi-automated methodology through ImageJ,^23^ endocrine cell quantification of ND and AAb+ donors using ImageJ,^24^ using random forest-based pixel classifier to segment of islets through QuPath,^25^ and a CNN model was harnessed to detect pancreatic islet regions.^26^

Instance segmentation^27^^,^^28^ which outlines each object in an image at the pixel level, has been widely and increasingly used in recent medical image analysis.^29^, ^30^, ^31^, ^32^ Segment Anything Model (SAM),^33^ a foundation image segmentation model trained by huge amount of data, is capable of identifying cellular objects with precise boundaries.^29^^,^^34^^,^^35^ SAM is a prompt-based segmentation model that comprises an image encoder, prompt encoder, and mask decoder. It processes prompts such as points, boxes, or text to generate the ground truth of the image and returns the highest-quality mask among the three generated masks. The artificial neural network (ANN)-based pixel classifier is a model used to classify individual pixels based on RGB vectors, allowing for the differentiation of the various cell types by analyzing the designated RGB stain values.

With the advancement of user-friendly pathology applications like the open-source QuPath,^36^ researchers are striving to expedite accurate islet and single cell analyses. QuPath offers diverse functions for analyzing bioimages, including cell detection and the ANN-based pixel classifier. Advanced deep learning models, such as Deep MIB,^37^ SAMapi,^34^ Stardist,^38^ WSInfer,^39^ can be utilized in the user-friendly interface, QuPath. It also provides the flexibility to craft customized scripts to facilitate more advanced processes.

**Fig. 1:**
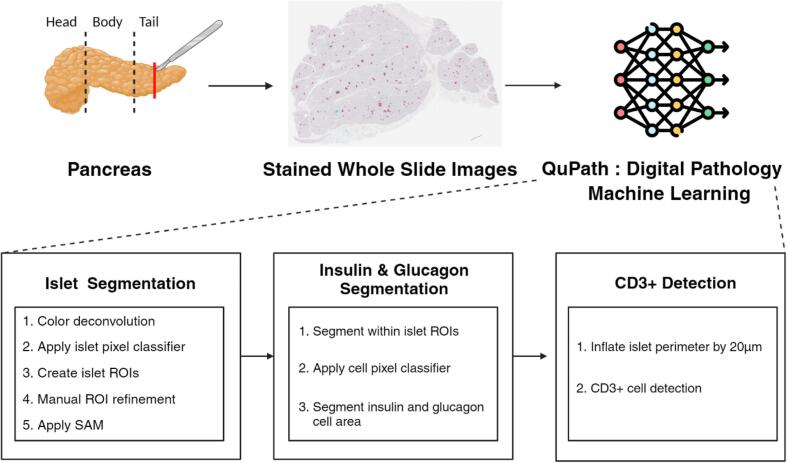
Overview of machine learning-assisted workflow using the user-friendly application QuPath. The main purpose of this approach is to efficiently quantify the number of endocrine cell groups and proportions of insulin, glucagon, and CD3+ cells within each of the segmented islets.

This work aimed to develop an enhanced machine learning-assisted WSI analysis workflow to analyze islet heterogeneity by leveraging the WSI data collected from nPOD. nPOD dataset is publicly available for studying objectives.^40^ The University of Florida's high-performance-computing resource known as HiPerGator aids in handling large sizes of the WSI data and using pre-trained deep learning models that can be used through QuPath. Fig. 1 illustrates how this developed workflow can be used to accelerate the analysis of pancreatic WSIs for islet segmentation, area segmentation of insulin and glucagon cells inside each islet, and CD3+ cell detection.

## Developed workflow

**Fig. 2:**
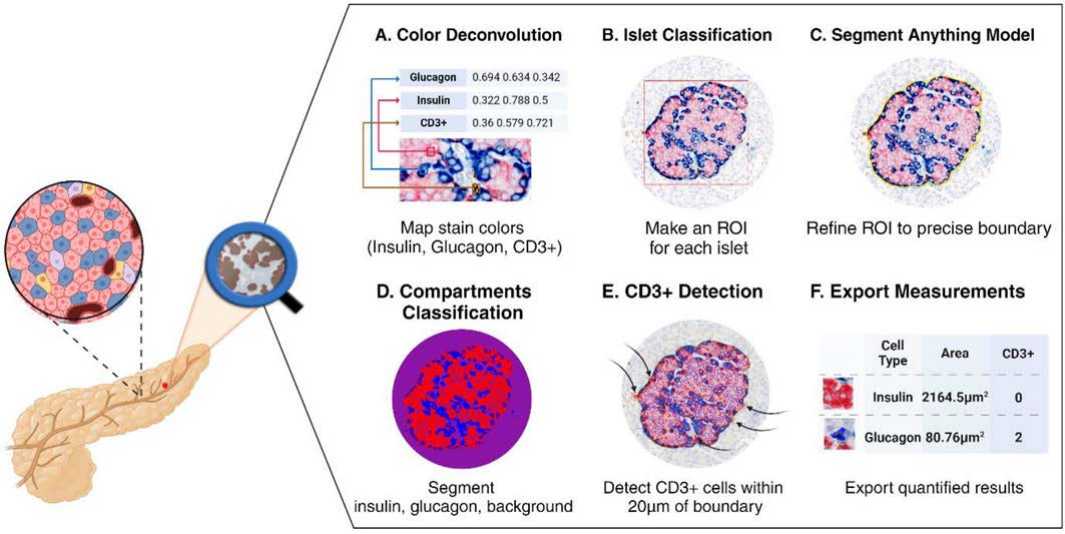
This outline demonstrates the proposed methodology using machine learning-assisted, semi-automated workflow. SAM is used to segment precise boundaries of endocrine cell groups, and the ANN-based pixel classifier is used to segment the insulin and glucagon areas.

QuPath (v0.4.4) was selected as the main interface for the developed workflow. Additional groovy scripts were developed to apply the inner capabilities of QuPath to streamline data processing. The deep segmentation model SAM trained by microscopy data^35^ through SAMapi (v0.5.0)^34^ and ANN-based pixel classifier provided the most accurate quantification of islet measurements. This combined methodology is crucial for analyzing pancreatic WSI images due to the diverse conditions of stain values under which data were collected. Additionally, a function implemented in QuPath, positive cell detection, was applied to detect the number of immune cells (CD3+ cells) around the segmented islets, considering the stain values of cells. Fig. 2 illustrates the developed methodology consisting of six steps. Each step of the developed methodology is described in more detail in the following sections, along with explanations of how to use the scripts provided as supplementary materials and case examples.

### Color deconvolution

Due to variability in the color stain spectrum across the brightfield WSI data (Fig. S1), we also allowed users to define RGB stain value separation for each image slide to enhance performance.^41^ Insulin, glucagon, and CD3+ cells were respectively stained in red, blue, and brown. Therefore, each slide varying in color designations leads to a wide range of stain values. To accurately define RGB stain values for each slide, users can run the Supplementary A1 script to change the identity of the three-color schemes to insulin, glucagon, and CD3+. Then, users can assign a square around each cell type in QuPath's image viewer to indicate the stain values for the corresponding cell type. Fig. 2A and Fig. S2a demonstrate the resulting stain values that fit each cell type in the particular image slide.

### Construction of ROI boxes

Specifying regions of interest (ROIs) around islets requires leveraging the pre-trained SAM with microscopy dataset^35^ for islet segmentation. The ROI can be constructed by applying an islet pixel classifier, which is implemented in QuPath as an ANN-based pixel classifier. This classifier is trained on each WSI individually, using a single islet segmented by pre-trained SAM and the background designated as an annotation square box. For training the islet pixel classifier, the segmented islet is created by drawing a rectangular box around the islet, followed by the application of SAM. The trained islet pixel classifier categorizes the classes of each cell within the value of RGB stains for each pixel and identifies the clustered islet and backgrounds. In addition, only clustered cells can be included in the analysis by defining the minimum object size (Fig. S2b). To reorganize the detected group into the islet, perspective of ROI boxes including insulin and glucagon, users can run the Supplementary A2 script after removing the backgrounds. This allows users to automatically construct the ROI boxes around numerous islets of various sizes and shapes. Fig. 2B and Fig. S2c illustrate the progression from candidate boxes to accurate red ROI boxes. To increase the accuracy of the islet analysis, users can further remove or adjust the ROI boxes manually in QuPath.

### Islet segmentation using SAM

For the accurate islet boundary quantification, the pre-trained SAM^35^ was applied to the ROI boxes. This SAM model is specifically designed for cell segmentation and it can be added to QuPath as an extension tool as SAMapi. By running SAM for all ROI boxes, the islet boundaries can be accurately defined. This process was accelerated through the use of HPG NVIDIA A100 graphics processing units (GPUs). Accurate islet segmentations using SAM are shown in Fig. 2C and Fig. S2c.

### Segmentation of insulin and glucagon

To quantify endocrine cells within islets, a cell pixel classifier was applied to classify three classes: insulin, glucagon, and background (Fig. 2D and Fig. S2d). The Supplementary A3 script was added to assign a number to each segmented islet (Fig. S2d). The cell pixel classifier detected areas of the insulin and glucagon classes for positive areas larger than 20 μm^2^*.* The resulting segmented areas of insulin and glucagon are shown in Fig. 2D and Fig. S2d.

### CD3+ cell detection

In this study, an islet was categorized as low T-cell infiltrated if there were one to six CD3+ cells, and as high T-cell infiltrated if there were more than six CD3+ cells either inside or within 20 μm of the islet perimeter. The 20 μm perimeter boundaries for each islet can be established automatically using the Supplementary A4 script (Fig. S2e). The Supplementary A5 script was developed to detect CD3+ cells using threshold setup by the mean and standard deviation of all cells in 20 μm boundary of each islet. Fig. 2E and Fig. S2f show the results of detecting CD3+ cells, annotated with a red doubled boundary.

### Export measurements

The quantified information can be exported using QuPath's built-in functions, such as annotation measurement and detection measurement. The exported measurements include the areas of insulin and glucagon, the number of CD3+ cells, and the diameter and circularity of each islet (Fig. 2F). The Supplementary A6 script was added to further process the extracted measurements to convert the QuPath exported text files to a single CSV format and extract the necessary data.

## Proof-of-concept analysis results

To demonstrate the developed workflow, IHC WSI files were obtained from the nPOD by file transfer. The average size of the WSI data was 43,234.5 × 33,600.958 pixels (W × H) with 0.497 μm per pixel. Table 1 summarizes donor demographics used for this proof-of-concept study using two slides from each pancreas tail region belonging to 12 donors. Four donors were selected from each of the disease groups: ND, AAb+, and T1D.

**Table 1:** Demographic summary. Two pancreas tail slides from each donor were analyzed, with four donors included per disease category.

Characteristics	ND	AAb+	T1D
	(*n* = 4)	(*n* = 4)	(*n* = 4)
Age, mean ± SD (years)	20.21 ± 14.53	22.78 ± 4.39	19.38 ± 9.35
Sex, *n*			
Female	0	3	1
Male	4	1	3
Ethnicity, *n*			
Caucasian	2	3	3
Hispanic	1	0	0
African American	1	1	1
BMI, mean ± SD (kg/m^2^)	20.65 ± 5.59	30.45 ± 12.58	20.60 ± 3.12
Diabetes duration, mean ± SD (years)	–	–	9.65 ± 11.34
C-peptide, mean ± SD (ng/ml)	4.56 ± 1.42	8.41 ± 2.90	0.04 ± 0.04
HbA1c, mean ± SD (%)	5.43 ± 0.63	5.38 ± 1.58	9.55 ± 2.28

SD: standard deviation, ND: no diabetes, AAb+: autoantibody-positive, T1D: type 1 diabetes, BMI: body mass index, HbA1C: hemoglobin A1C.

**Fig. 3:**
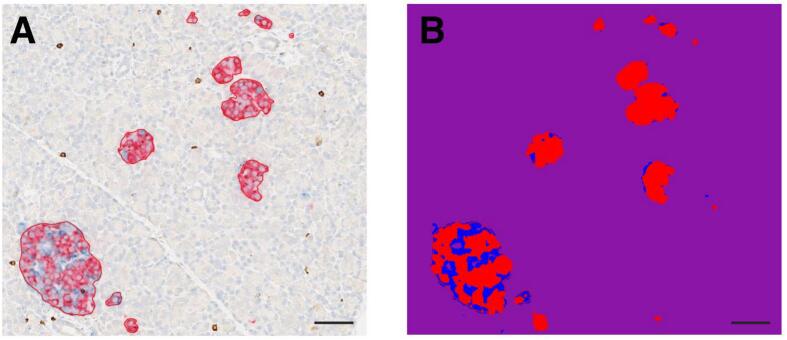
Representative results demonstrating: (A) the boundaries of endocrine cell groups segmented by SAM, (B) the three classes (i.e., insulin (red), glucagon (blue), and background (purple)) segmented using the ANN-based pixel classifier. (Scale bar = 50 μm.)

As shown in Fig. 3A, the advantage of utilizing SAM is the accurate segmentation of endocrine cell boundaries. The quality score, representing the intersection over the union (IoU) (Eq. 1) metric, which quantifies the overlap between the segmentation mask and the ground truth generated by the mask decoder and image encoder, is displayed above each segmented islet. SAM demonstrates the highest-quality segmentation mask among the three candidate masks. All islets were segmented with a high quality score (i.e., an average quality score of 0.91 per slide) in the proof-of-concept analysis. Fig. 3B demonstrates the performance of the cell pixel classifier which segments the endocrine cell compositions into insulin and glucagon.

(1) $$\mathrm{IoU}=\frac{\text{Mask}\cap \text{Ground Truth}}{\text{Mask}\cup \text{Ground Truth}}\;$$

**Fig. 4:**
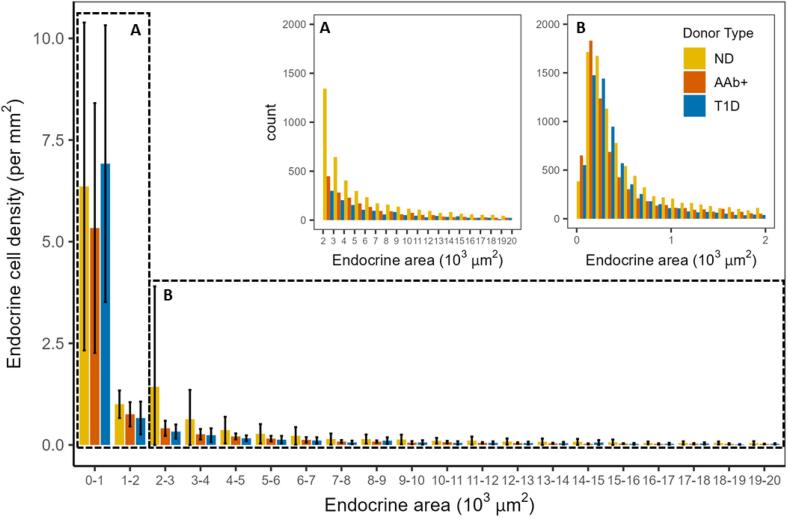
The proposed methodology effectively detects the diverse heterogeneity of endocrine cell groups, including single cells. The bar graph illustrates the mean and standard deviation of the number of endocrine cell groups per pancreas slide categorized by size. The endocrine cell groups vary in size ranging from single endocrine cells to endocrine cell groups. (A) Shows the count of the endocrine cell groups per area (endocrine cell group size: 0–2000 μm^2^), (B) shows the count of endocrine cell groups per area (endocrine cell group size: 2000–20,000 μm^2^).

Fig. 4 shows the use of SAM and ANN-based pixel classifiers to enable the identification of all endocrine cells in the pancreas WSIs ranging from single endocrine cells to large islets. We have found that the majority of components of the pancreas tissue are small endocrine single cells and clusters. Our methodology has been able to successfully identify around 10,000 additional endocrine cells using our semi-automated workflow with delicate segmentation.

**Fig. 5:**
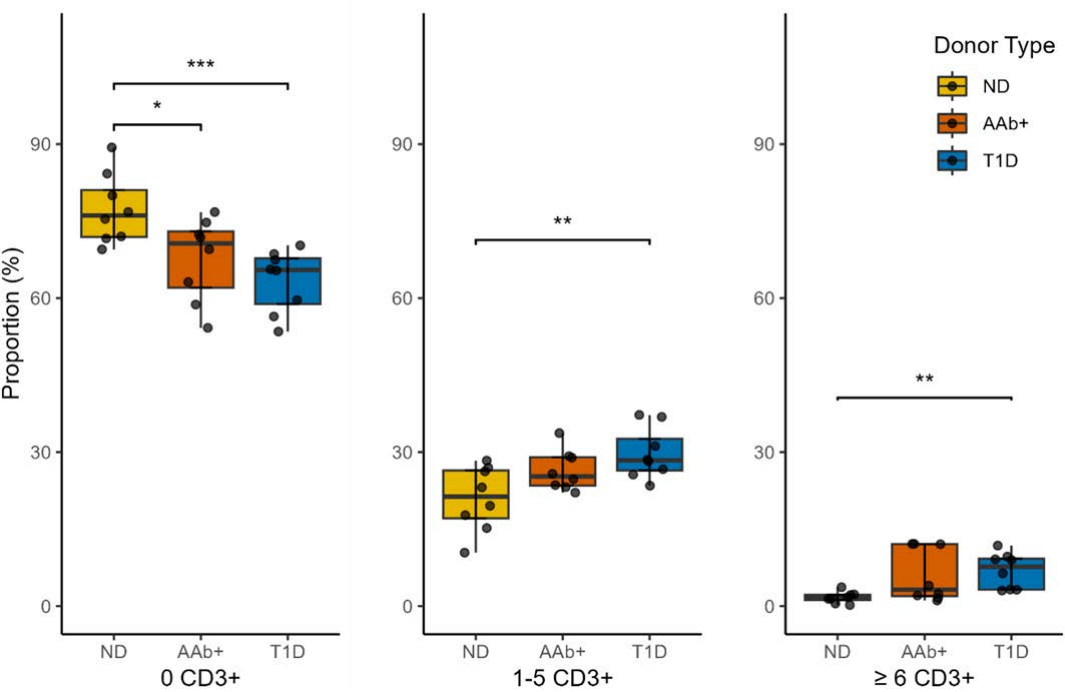
The proportion of endocrine cell groups without CD3+ cells, containing 1–5 CD3+ cells, and containing more than or equal to 6 CD3+ cells inside and within the 20 μm perimeter among all detected endocrine cell grouping. Each dot represents a tail slide from a single donor. The Wilcoxon test is applied, and non-significant (NS) results are not shown in the graphs. (*p*-values, ***: *p* < 0.001, **: *p* < 0.01, *: *p* < 0.1). Abbreviations: ND: no diabetes, AAb+: autoantibody-positive, T1D: type 1 diabetes.

In addition, the scripts of our methodology assist in detecting the CD3+ cells around the segmented endocrine single cells, clusters, and islets. The exact quantification of the number of CD3+ cells around the islets is important for identifying CD3+ T-cell infiltration. Fig. 5 demonstrates that ND donors have around 77.3% islets without CD3+ cells, 21.6% with 1–5 CD3+ cells, and 1.1% with ≥6 CD3+ cells. Notably, none of the ND donors had insulin-negative islets, therefore, CD3+ T-cell infiltration is not considered in ND donors. In AAb+ donors, these percentages are 67.7%, 27.8%, and 4.5%, whereas in T1D donors, the values are 63.4%, 31.0%, and 5.6%, respectively.

## Discussion

WSI analysis contributes to expediting and facilitating the analysis of donors with ND, AAb+ presence, and T1D. Previous work applying WSI analysis to pancreatic tissue, especially manual digital analysis, identified several challenges. Biased interpretation and inter-observer variability occur even when analyzing identical data due to the variety of stained color values in the WSIs. The adaptability of stained color values from the initial stage of the proposed methodology, specifically the color deconvolution, makes the analysis more robust to the diverse staining patterns encountered on slides. In addition, compared to previous WSI analyses, our proposed semi-automated methodology enables the precise extraction of islets, even small endocrine cells, with accurate boundary delineation. Furthermore, it facilitates the extraction of key information from each slice, including islet diameter, insulin and glucagon measurements within each islet, and the number of CD3+ cells within a designated perimeter boundary. The results of our analysis demonstrate the accuracy of our methodology and could provide insights into the potential discovery of new characteristics of T1D.

We designed our approach as a solution to support analysts who may not have extensive experience with digital analysis. Each script encompasses a range of functions designed to execute specific tasks, allowing users to analyze WSI data without needing to navigate complex software interfaces. Notably, the proposed scripts are not limited to workflow execution but also aid in post-processing data to streamline analysis procedures and assist with statistical analysis. Furthermore, the proposed methodology can be adapted for widespread applicability in the analysis of various diseases by developing or modifying the proposed scripts, leveraging the extensibility of QuPath.

Using advanced deep learning and high-performance-computing resources, our methodology enables a more detailed and non-biased analysis. Unlike previous analyses, our methodology detects various sizes of ranging from single endocrine cells to islets. Additionally, it enhances the performance of extracting islets from WSIs through the combination of the deep segmentation model SAM and ANN-based islet pixel classifier. The additional manual modification of ROI boxes by users can also improve accuracy and increase confidence in islet segmentation. Nonetheless, this manual step offers users the flexibility to adjust and validate the ROIs as needed, ensuring precision in the segmentation process.

Based on the precise segmentation of islets, the quantification of the islet area is likely more accurate than prior methods, suggesting a new standard for islet definition in terms of cell size. With our methodology, users can quantify the size of a single cell and determine the area of an islet. Additionally, well-segmented islets facilitate the measurements of insulin and glucagon within the islet, which are quantified using the cell pixel classifier at high resolution. The accurate assessment of the areas of insulin and glucagon can be used as new analysis criteria for the study of T1D in WSIs.

The detection and quantification of the number of CD3+ cells around the islet or single endocrine cell are accurately performed through our methodology. This approach broadens the opportunity to analyze T1D pathology and understand the pathogenesis of T1D. Hence, our methodology provides the flexibility to define CD3+ T-cell infiltration based on CD3+ cells. Although, previous studies have defined CD3+ T-cell infiltration by islets containing a certain number of CD3+ cells, users can now modify the definition of CD3+ T-cell infiltration through our proposed scripts. These scripts will set a new perimeter boundary and new CD3+ T-cell infiltration standard for the number of CD3+ cells around a single islet.

From a practical use standpoint, our methodology accelerates the analysis time with the assistance of the user-friendly application QuPath and deep learning. Based on our observations, the time needed per slide ranged from 40 min to 1.5 h, influenced by factors such as computer resources and the quantity of islets within each slide.

Our semi-automated T1D pathology methodology can contribute to the accurate quantification of T1D-related WSI analysis in donors. Particularly in quantifying islets, identifying the ratio of insulin and glucagon within individual islets, and the number of CD3+ cells around the islets. Through this methodology, trends or characteristics of T1D progression are expected to be analyzed based on quantified information from various nPOD donors. Furthermore, analyzing the phenomenon through the pancreas WSI will be more accurate and faster compared to manual analysis, making it extensively useful. Ultimately, the methodology will be applicable for analysts because it is well organized with open-source programs.

As demonstrated by the results of the experiment, the developed methodology was applied to only a limited number of WSIs from each donor, which is insufficient for detailed analysis. Analyzing a large number of WSIs from various donors is necessary for understanding the pathogenesis of T1D. Our methodology can be harnessed to the diverse WSIs available through the assistance of biobanks, which offer a large quantity of accurate, and high-resolution data. It is expected that various new findings will be revealed through detailed analysis.

Processing time varies depending on computer resources and individual factors. The analysts spent the majority of time completing manual ROI modifications and running the scripts for detecting CD3+ cells. Manual ROI adjustments are subject to individual variability, whereas the performance of SAM relies on GPU capabilities and the quantity of ROIs, and CD3+ cell detection is influenced by CPU performance.

In our future work, we plan to leverage our analysis workflow with the WSI data from nPOD to further accelerate the understanding of T1D pathogenesis. A large set of WSIs from multiple donors and pancreatic regions (head, body, and tail) will be analyzed using our workflow. In particular, the ability to quantify features of small endocrine cells and groups, alongside detailed quantification of insulin and glucagon areas, will provide new insights into the understanding of T1D.

## Conclusion

The machine learning-assisted WSI analysis workflow was developed to analyze islet heterogeneity to add to current T1D research. To increase usability, the user-friendly open-source software QuPath was used as the main interface. The SAM pre-trained with microscopy dataset was employed to define precise islet boundaries and the cell pixel classifier was applied to segment the areas of insulin and glucagon within each islet. An additional script was developed to count CD3+ cells inside and within 20 μm of each islet perimeter to quantify the number of islets with inflammation (i.e., CD3+ T-cell infiltration). Proof-of-concept analysis was performed to test the developed workflow. This machine learning-assisted methodology enables identifying precise islet boundaries and cell compositions for endocrine cell clusters of all sizes, including those smaller than islets. Our contribution to the field of T1D research will accelerate the analysis of pancreas slides and provide insights into better understanding of islet heterogeneity.

## Author contribution

S. Kang, M. Campbell-Thompson, and S. Kim wrote the manuscript.

J. Penaloza Aponte, M. Campbell-Thompson, and S. Kim designed the research.

S. Kang, J. Penaloza Aponte, O. Elashkar, J.F. Morales, N. Waddington, and S. Kim performed the research.

S. Kang, D. Lamb, H. Ju, M. Campbell-Thompson, and S. Kim analyzed the data.

## Funding information

This work was supported by Breakthrough T1D (3-SRA-2022-1157-S-B) and NIDDK R01DK123329 and U54DK127823.

## Declaration of competing interest

The authors declare the following financial interests/personal relationships which may be considered as potential competing interests:

Sarah Kim reports financial support was provided by Breakthrough T1D (3-SRA-2022-1157-S-B). Martha Campbell-Thompson reports financial support was provided by The National Institute of Diabetes and Digestive and Kidney Diseases (R01DK123329). Martha Campbell-Thompson reports financial support was provided by The National Institutes of Health (U54DK127823). Sarah Kim & Martha Campbell-Thompson reports a relationship with Network for Pancreatic Organ Donors with Diabetes that includes: non-financial support. Sarah Kim reports a relationship with NVIDIA AI Technology Center at University of Florida that includes: non-financial support. If there are other authors, they declare that they have no known competing financial interests or personal relationships that could have appeared to influence the work reported in this article.

## Appendix A Supplementary data

Supplementary material The supplementary materials provide a detailed workflow and code explanation. Supplementary material
