# Supplementary material for: Leveraging pre-trained machine learning models for islet quantification in type 1 diabetes
Source: J Pathol Inform. 2024 Nov 8;16:100406. doi: 10.1016/j.jpi.2024.100406 (PMC11665367; doi:10.1016/j.jpi.2024.100406)
Supplement: Supplementary material — The supplementary materials provide a detailed workflow and code explanation. [file mmc1.docx]

**Supplementary Materials**

**A**. Groovy scripts for QuPath (1-5) and postprocessing script using Python (6)

| **1. Set Color Deconvolution**  */**  *The values for each stain are pre-set for*   - *Glucagon (blue)* - *Insulin (red)* - *CD3+ (brown)*   *Warning: replace these values accordingly*  **/*  setColorDeconvolutionStains("""{  "Name" : "HE_Insulitis",  "Stain 1" : "Glucagon",  "Values 1" : "0.694 0.634 0.342",  "Stain 2" : "Insulin",  "Values 2" : "0.322 0.778 0.54",  "Stain 3" : "CD3+",  "Values 3" : "0.38 0.579 0.721",  "Stain 4" : "Tissue",  "Values 4" : "0.38 0.579 0.721",  "Background" : "255 255 255"  }"""); |
| --- |

| **2. Convert ROI box**  *// Select ROIs and make bounding boxes*  *// Stores all the annotation objects product of running color segmentation in a variable*  rois = getAnnotationObjects().collect{it.getROI()}  *// Iterates over each ROI*  rois.each {  *// Defines a bounding box with a preset pixel value*  boundingROI = ROIs.createRectangleROI(  it.getBoundsX() - move_left_corner, // top left corner  it.getBoundsY() - move_left_corner, // top left corner  it.getBoundsWidth() + expand_box, // width  it.getBoundsHeight() + expand_box, // height  plane)  *// Stores each bounding box in a list*  boundingAnnotation << PathObjects.createAnnotationObject(boundingROI)  } |
| --- |

| **3. Rename ROI annotation into ‘Islet’**  *// Rename each annotation following SAM segmentation*  def baseName = "islet" // name  def counter = 1 // count  *// Iterate over each annotation and update the name with the prefix and the counter value*  annotations.each { it ->  def newName = "${baseName}_${counter}"  it.setName(newName)  counter++  } |
| --- |

| **4. Expand Boundary of Islet**  *// Expands the boundary of the previously define islet to 20.0um*  double expandMarginMicrons = 20.0 // Set value in um to expand the islet *// Converts microns to pixel values*  double expandPixels = expandMarginMicrons / cal.getAveragedPixelSizeMicrons()  isletsList.each{ it ->  *// Gets the name of the iterated annotation*  def baseName = it.getName()   *// Generates the new name for the expanded islet*  def newName = (baseName ? "${baseName}_$suffixName" : suffixName)  *// Extracts the geometry object*  currentArea = it.getROI().getGeometry()  *// Expands the area of the islet by the predefine pixel to microns value*  areaExpansion = currentArea.buffer(expandPixels)  *// Converts the geometry to ROI*  roiExpansion = GeometryTools.geometryToROI(areaExpansion, plane)  *// Converts the ROI to an annotation*  annotationExpansion = PathObjects.createAnnotationObject(roiExpansion,  getPathClass("IsletExpanded"))  *// Sets the new name of the annotation*  annotationExpansion.setName(newName)  *// Stores the new annotation in the list*  annotationToAdd << annotationExpansion  } |
| --- |

| **5. Detect CD3+ cells**  *// Calculate mean and standard-deviation of the intensity feature of cells inside the expanded boundary*  double overallMean = totalMean / count  double overallStdDev = totalStdDev / count  double cd3Threshold = overallMean + ((overallStdDev))  *// Run function of Positive Cell Detection*  annotationsIsletExpanded.each { it ->  getCurrentHierarchy().getSelectionModel().setSelectedObject(it, false)  runPlugin('qupath.imagej.detect.cells.PositiveCellDetection', jsonParams) } |
| --- |
| **6. Pre-processing Measurement Data**  *# Calculate mean and standard-deviation of the intensity feature of cells inside the expanded boundary*  *# Filter the area of Glucagon & Insulin = 0*  read_organized_file = os.path.join(organized_path, files)  quan_df = pd.read_csv(read_organized_file)  filtered_index = quan_df[(quan_df['Glucagon'] == 0) & (quan_df['Insulin'] == 0)].index  quan_df.drop(filtered_index, inplace=True)    quan_df2 = quan_df.drop(quan_df.columns[0],axis=1)  quan_df2.to_csv(f'{save_path}/organized_{files}')  *# Extract information from ‘Annotation’ and ‘Detection’ csv files*  areas_header = ['Class', 'Parent', 'Area µm^2', 'Length µm',  'Max diameter µm', 'Min diameter µm']  cd3_header = ['Name', 'Num Positive']  *# Save ['islet_name', num_cd3+, glucagon_area, insulin_area] -> excel*   results_df = pd.DataFrame(padded_numpy_combined_cd3,   columns=['Name', 'Num Positive', 'Glucagon', 'Insulin',  'Background', 'Length', 'Max_diameter','Min_diameter']) |

| 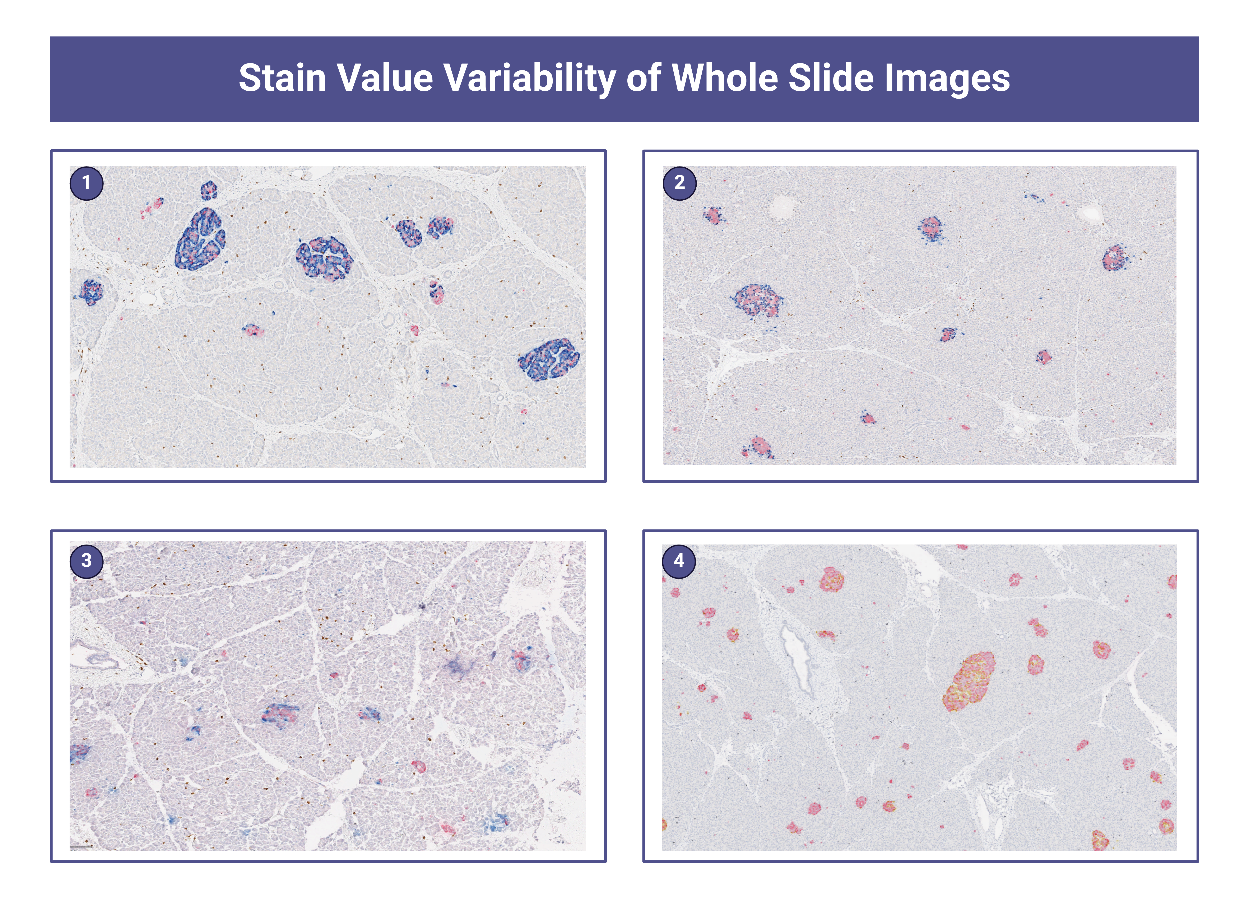  **Figure S1**. The sample slides with different color stain values. Some slides exhibit slight variations in color values, while others display entirely different color values. |
| --- |
| 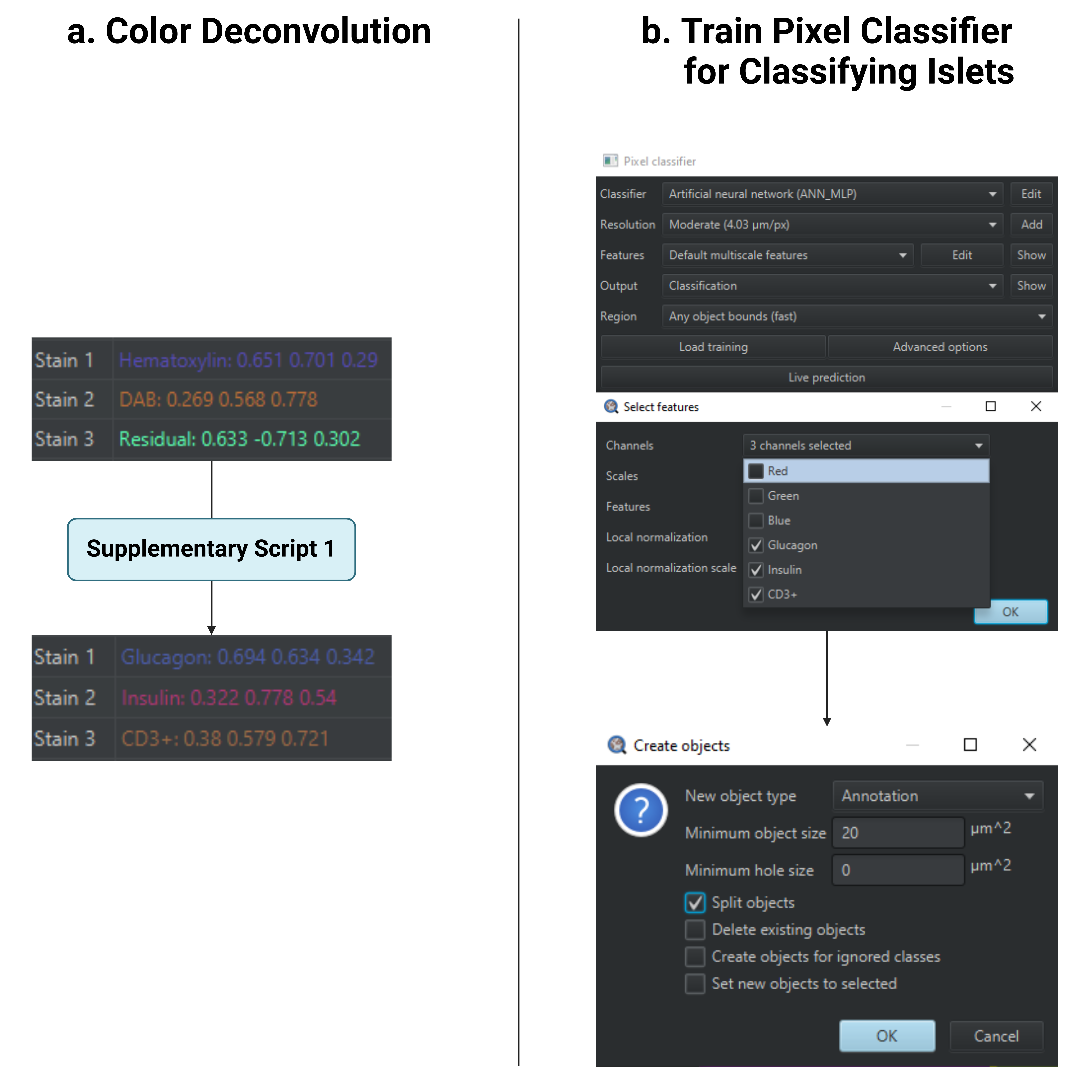  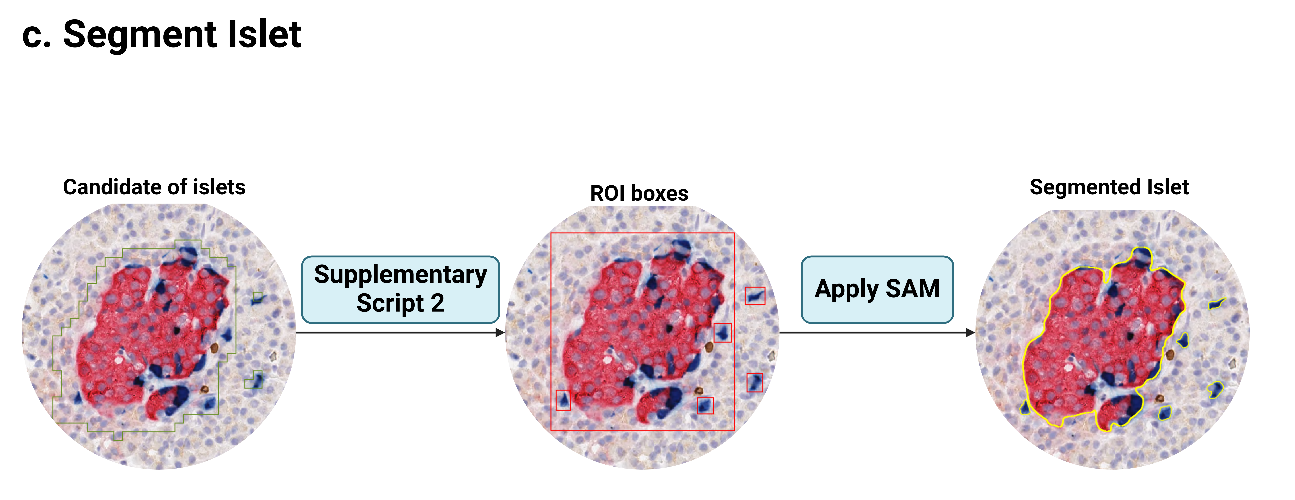  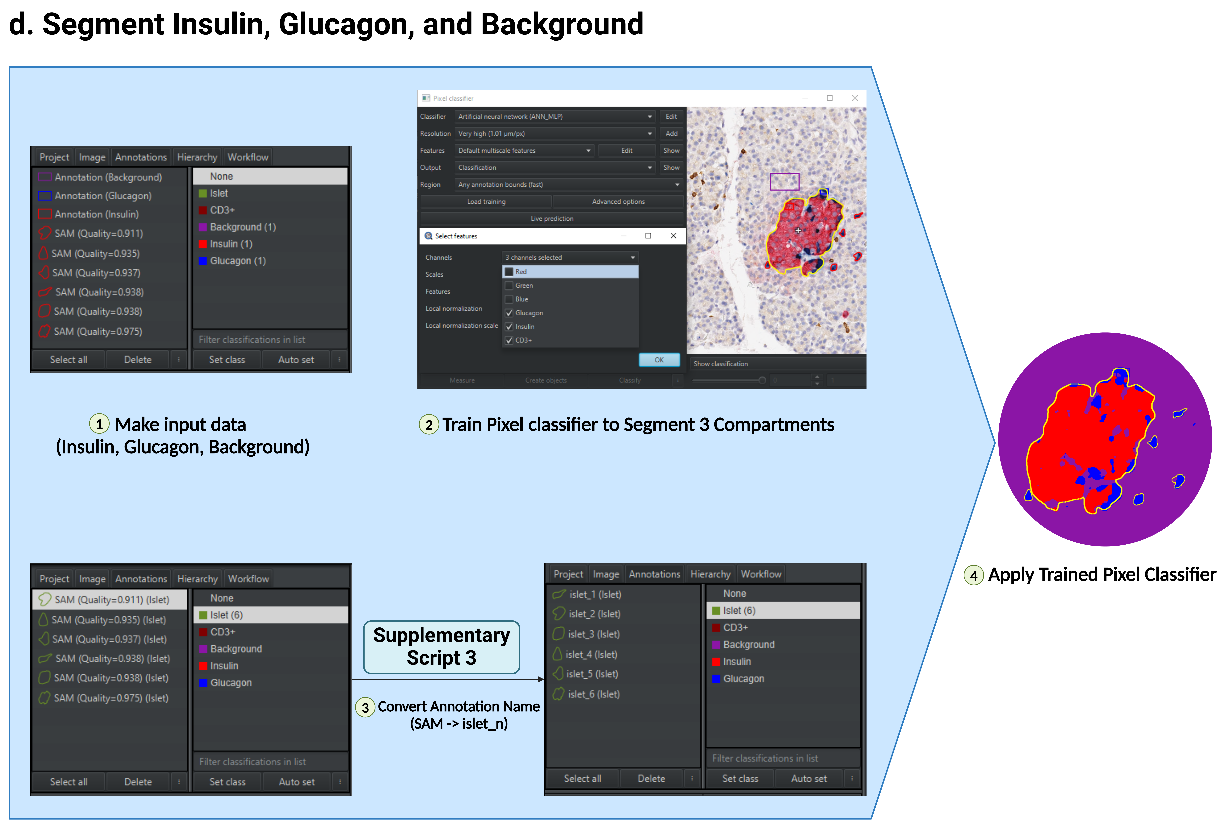  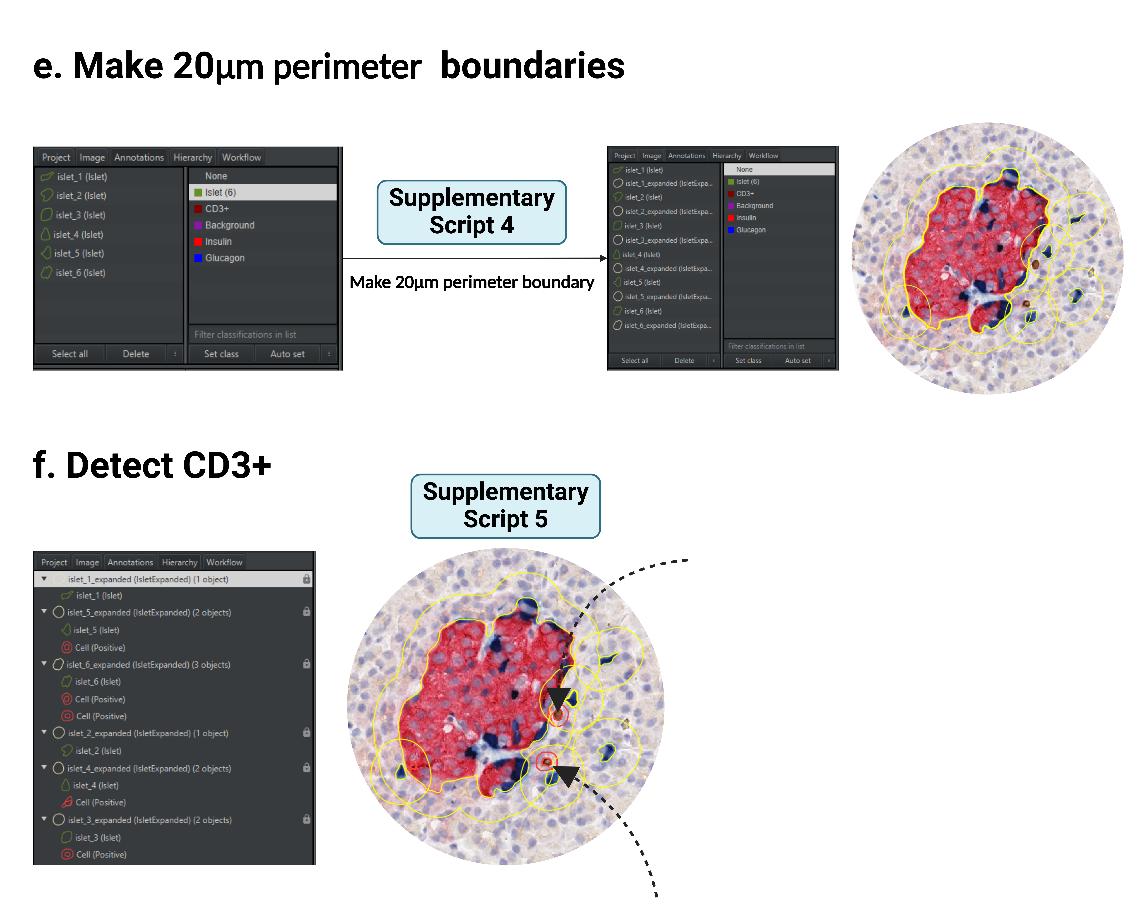  **Figure S2**. Detailed procedures of how to organize the islet segmentation workflow utilizing SAM, pixel classifier, and scripts with the following specific parameters. |
